# Elevated carboxylesterase activity contributes to the lambda-cyhalothrin insensitivity in quercetin fed *Helicoverpa armigera* (Hübner)

**DOI:** 10.1371/journal.pone.0183111

**Published:** 2017-08-17

**Authors:** Chengyu Chen, Ying Liu, Xueyan Shi, Nicolas Desneux, Peng Han, Xiwu Gao

**Affiliations:** 1 Department of Entomology, College of Plant Protection, China Agricultural University, Beijing, China; 2 INRA (French National Institute for Agricultural Research), Université Nice Sophia Antipolis, CNRS, UMR 1355–7254, Institut Sophia Agrobiotech, Sophia Antipolis, France; Institute of Zoology Chinese Academy of Sciences, CHINA

## Abstract

Quercetin as one of the key plant secondary metabolite flavonol is ubiquitous in terrestrial plants. In this study, the decrease in sensitivity to lambda-cyhalothrin was observed in quercetin*-*fed *Helicoverpa armigera* larvae. In order to figure out the mechanisms underlying the decreased sensitivity of *H*. *armigera* larvae to lambda-cyhalothrin by quercetin induction, the changes in carboxylesterase activity and in-vitro hydrolytic metabolic capacity to lambda-cyhalothrin were examined. The LC_50_ value of quercetin-fed *H*. *armigera* larvae to lambda-cyhalothrin showed 2.41-fold higher than that of the control. S, S, S-Tributyl phosphorotrithioate (DEF) treatment showed a synergism effect on lambda-cyhalothrin toxicity to quercetin-fed *H*. *armigera*. Moreover, the activity of carboxylesterase was significantly higher in quercetin-fed *H*. *armigera* larvae after fed on quercetin for 48 h. The in-vitro hydrolytic metabolic capacity to lambda-cyhalothrin in quercetin-fed *H*. *armigera* larvae midgut was 289.82 nmol 3-PBA/mg protein/min, which is significant higher than that in the control group (149.60 nmol 3-PBA/mg protein/min). The elevated CarE enzyme activity and corresponding increased hydrolytic metabolic capacity to lambda-cyhalothrin in quercetin-fed *H*. *armigera* contributed to the enhanced tolerance to lambda-cyhalothrin.

## Introduction

In plant-insect interactions, some plant secondary metabolites exert the defensive roles by interfering basic metabolic, biochemical, physiological functions of herbivorous insects [1]. Many kinds of plant secondary metabolites have been shown resistant to the herbivorous insects from several orders: Coleoptera, Lepidoptera, Hymenoptera and Hemiptera, by acting as feeding deterrents, growth inhibitors or toxins [2–5].

However, insects do not act as passive victims. It has been extensively documented that detoxification enzyme systems in insects played key roles in coping with plant defensive secondary metabolites [6–8]. Besides cytochrome P450 monooxygenases (P450s) in insects can detoxify plant secondary metabolites [9], carboxylesterase (CarE), as one of the important detoxification enzymes in insects, is also known to be induced by plant secondary metabolites. For example, CarE activity is significantly induced in *Lymantria dispar* after being exposed to phenolic glycoside [10]. A higher CarE activity was found in *Sitobion avenae* which fed on high indole alkaloid content during vegetative growth of wheat [11].

The detoxification enzyme system of insects that involved in detoxification metabolism of plant secondary metabolites also could metabolize and detoxify insecticides. Thus, the changes in insect detoxification enzyme activity in response to plant secondary metabolites may result in variations in insecticides sensitivity. It is known that the sensitivity of insect to insecticide could be affected by pre-treating with plant secondary metabolites. For example, when the generalist two-spotted spider mite *Tetranychus urticae* shifts host plant from their common host *Phaseolus vulgaris* (kidney beans) to a more challenging and less accepted host *Solanum lycopersicum* (tomato), the insecticidal activity of acaricide decreased due to different characteristics of secondary metabolites in the two host plants [12]. Cross-resistance to α-cypermethrin after xanthotoxin ingestion was also observed in *Helicoverpa zea* (Lepidoptera: Noctuidae) [13]. Quercetin, as plant secondary metabolite flavonol, is ubiquitous in terrestrial plants. In addition to the deleterious effects of quercetin on the development and survival of *H*. *armigera* [14,15] and other lepidopterous insects [16, 17], the effect of dietary quercetin on the CarE activity in silk worm was also observed [18]. However, the influence of quercetin on insecticides sensitivity and related metabolic capacity of insects has been rarely documented.

To examine how the insecticide sensitivity of insects is affected by oral exposure to host plant secondary metabolites [19, 20], we investigate the effects of quercetin intake on the *H*. *armigera* larvae sensitivity to lambda-cyhalothrin. *H*. *armigera* is one of the most important polyphagous pest insect, which attacks more than 200 plant species throughout the world. Thus, various plant secondary metabolites including quercetin were inevitably ingested by *H*. *armigera* through feeding on various host plants, such as cotton and *solanaceous* vegetable etc [21, 22]. Meanwhile, many insecticides including pyrethroid insecticide lambda-cyhalothrin are still widely used to control *H*. *armigera* [23]. Therefore, clarifying the effects of quercetin intake on *H*. *armigera* larvae sensitivity to lambda-cyhalothrin is important for the IPM of this pest insect.

## Materials and methods

### Insects

The cotton bollworm *H*. *armigera* (Hübner) colony was built by collecting the adults from Handan, Hebei Province, China, permitted by the local agricultural sector. They were reared on an artificial diet without exposure to any insecticides for more than 70 generations, in a conditioned room maintained at 25±1°C, 70–80% relative humidity, with a 16:8 (L:D) photoperiod. Adults were held under the same conditions and supplied with a 10% sugar solution. The rearing method was referred to Liu *et al* [24].

### Chemicals

Quercetin, α-Naphthyl acetate (α-NA), Tributyl phosphorotrithioate (DEF) and fast blue B salt were obtained from Sigma-Aldrich. Lambda–cyhalothrin was obtained from Shanghai Chemical Reagent Company with greater than 98% purity. Triton X-100 was from Amresco. 3-Phenoxybenzoic acid (3-PBA) was from Alfa Aesar. All other chemicals and solvents used were analytical reagent grade.

### Bioassays and synergism experiment

A leaf-dipping bioassay was used to evaluate the toxicity of lambda–cyhalothrin to the third-instar larvae of *H*. *armigera* [25].

The effects of quercetin intake on lambda-cyhalothrin sensitivity of *H*. *armigera* larvae were assayed using the following protocols. The treatment group of *H*. *armigera* larvae were fed on artificial diet incorporated with 0.1% quercetin (g/g artificial diet) for 48 h, while the control group fed on the artificial diet without quercetin. Lambda-cyhalothrin was dissolved in acetone and diluted to a series of concentrations (150, 180, 210, 240 and 270 mg/L for treatment group; 60, 85, 110, 135 and 160 mg/L for control group) with distilled water containing 0.1% Triton X-100. Cabbage *Brassica oleracea L*. leaf was cleaned using distilled water and cut into discs. The cabbage leaf discs were dipped into the above-mentioned insecticide solutions for 10 s and placed in shade to air dry, and then transferred to 12-well tissue-culture plates containing 2% agar covered with filter paper. Bioassay was carried out by inoculation of 36 third instar larvae of *H*. *armigera* to lambda-cyhalothrin treated leaves for each concentration (12 per well and three replicates for each concentration). The control was treated with leaves dipped in distilled water containing 0.1% Triton X-100 and 1% acetone.

Synergist DEF can inhibit the activity of carboxylesterase in insect [26]. Insecticide toxicity in the presence or absence of synergist DEF was evaluated on both the quercetin-fed and the control *H*. *armigera* larvae described above. DEF was dissolved in acetone, and 12 μg of DEF solution was topically delivered onto the prothorax notum of each *H*. *armigera*. After 4 h, the DEF-treated *H*. *armigera* larvae were used for evaluating the toxicity of lambda–cyhalothrin (80, 95, 110, 125 and 140 mg/L for quercetin-treated group; 40, 50, 60, 70 and 80 mg/L for control group). The synergism was determined to be significant (P ≤0.05) when the 95% CLs for the LC_50_ values for the treatment with insecticide alone did not overlap with those for the treatment with synergist and insecticide [27]. The synergism ratio was calculated by dividing the LC_50_ value of insecticide alone by the LC_50_ value of insecticide with a synergist. Each experiment was repeated in triplicates. The mortality of bioassays and synergism experiment were assessed after lambda–cyhalothrin application for 48h.

### Carboxylesterase enzyme activity assay

The third-instar larvae of *H*. *armigera* with uniform size starved for 4 h and then transferred to artificial diet which incorporated with 0.1% (g/g artificial diet) quercetin.

The midguts of *H*. *armigera* were collected and then used for carboxylesterase enzyme activity assay for both quercetin and control group at 12, 24, 48, 72, and 120 h, respectively. The carboxylesterase enzyme activity of *H*. *armigera* larvae midguts was assayed by using the method described previously with some modification [24]. Briefly, the midguts of two-day-old third instar larvae of *H*. *armigera* were obtained by dissection on ice. The midguts was gently shaken to free of its contents and rinsed in an ice-cold 1.15% (m/v) potassium chloride aqueous solution. The homogenization buffer for CarE assay was phosphate buffer (0.04 M, pH 7.0). Ten midguts of *H*. *armigera* larvae were homogenized on ice with 1.5 mL of homogenization buffer, and then centrifuged at 10,800g for 20 min at 4°C. The homogenate was collected for CarE assay.

Carboxylesterase activity of these midgut homogenates was determined with *α*-naphtyl acetate (*α*-NA) as the substrate [28]. The enzyme reaction mixture for CarE activity assay contained 50 μL of enzyme preparation, 450 μL of 0.04 M phosphate buffer at pH 7.0, and 3.6 mL of *α*-NA solution (0.3 mM). The reaction was terminated by adding 0.9 mL of stop solution (two parts of 1% fast blue B and five parts of 5% sodium dodecyl sulfate) after incubation at 30°C for 15 min. The color was allowed to develop for another 15 min at room temperature, and the absorbance of the hydrolysis product, α-naphthol, was measured at 600 nm. Each sample was analyzed in triplicates. Determination of protein concentration was carried out using bovine serum albumin as the standard protein [29].

### In-vitro hydrolytic metabolism of lambda–cyhalothrin by *H*. *armigera* larvae midgut homogenate

3-Phenoxybenzoic acid (3-PBA), as a major metabolite of 3-phenoxybenzyl pyrethroids or *α*-cyano-3-phenoxybenzyl pyrethroids, the metabolism of cypermethrin catalyzed by crude homogenates of both rat and human liver microsomes, and the metabolism of *β*-cypermethrin by *H*. *armigera* homogenates were successfully evaluated by quantifying the production of 3-PBA [30, 31]. Hence, the in-vitro metabolism of lambda-cyhalothrin in midgut homogenates of *H*. *armigera* larvae via hydrolytic metabolism system was investigated by quantifying the production of 3-PBA.

Thirty midguts of *H*. *armigera* larvae were homogenized, on ice, in 3 mL of homogenization buffer (0.04 M phosphate buffer at pH 7.0). The homogenate was centrifuged at 4°C, 10800 g for 15 min. The supernatant was filtered through glass wool and collected into a clean ice-cold Eppendorf tube, and used immediately for in- vitro lambda–cyhalothrin metabolism assay.

**Fig 1:**
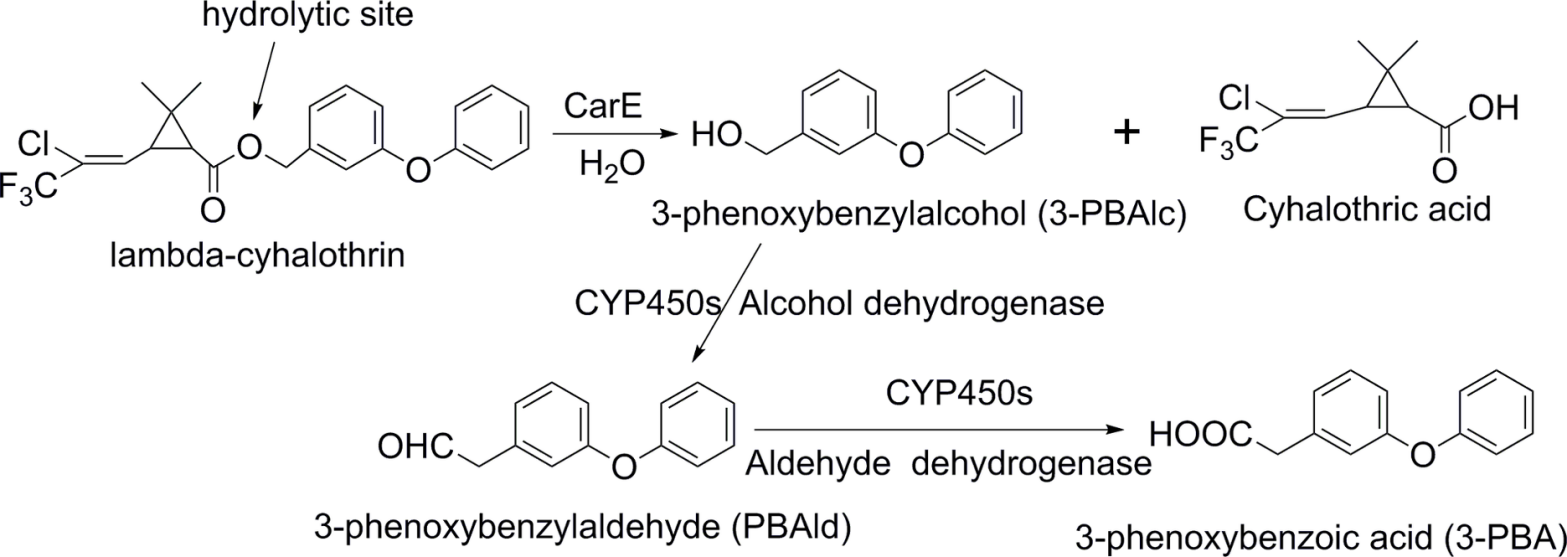
The metabolic pathways of lambda–cyhalothrin.

The in-vitro hydrolytic metabolism reactions of lambda-cyhalothrin catalyzed by the homogenates of *H*. *armigera* larvae midguts was performed in a total volume of 2 mL at 30°C for 120 min in a water bath with occasional shaking. The incubation mixture consisted of 0.04 M sodium phosphate buffer at pH 7.0 and 0.1 mM lambda–cyhalothrin. After the reaction mixtures were pre-incubated for 5 min, metabolic reactions were initiated by adding 0.5 mL of enzyme preparation (about 2 mg of proteins). After 120 min incubation, metabolic reactions were terminated by extracting with 2.5 mL mixture of ice-cold ethyl acetate/n-hexane (2:1, v/v) containing 0.1% phosphoric acid. Another 1.5 mL and 1 mL of ice cold ethyl acetate/n-hexane (2:1) mixture was added to extract the remaining metabolites, respectively. The organic fraction of three extracts was combined together and evaporated to dryness under a gentle nitrogen stream. The residue was re-dissolved in 200μL of acetonitrile and a 20-μL filtered solution was injected for High Performance Liquid Chromatography (HPLC) analysis. Metabolic reactions were performed in triplicates. Additionally, control incubations (without enzyme samples) and blank incubations (without substrates) were prepared in order to differentiate between metabolites originating from the enzyme samples and possible metabolites from the incubation procedure. Determination of protein concentration was done as described above. The metabolic pathways of lambda–cyhalothrin were shown in Fig 1.

### High performance liquid chromatography (hplc) analysis system

HPLC-DAD analysis of lambda–cyhalothrin and its metabolites was performed on an Agilent 1100 HPLC system (Agilent Company, USA) combined with a quaternary pump, online degasser, diode array detector (DAD), 7725i injection valve equipped with a 20-μL loop, and column thermostat, using a ZORBAX SB-C18 column (250 mm×4.6 mm i.d., 5μm, Agilent, USA). The mobile phases used were solvent A (acetonitrile), B (methanol), and C (H_2_O, adjusted to pH 2.1 with 85% phosphoric acid). The analytes were eluted with the following gradient program (linear increase): 0 min (0% A, 5% B, 95% C), 15 min(37% A, 5% B, 58% C), 25 min (60% A, 5% B, 35% C), 50 min (85% A,5% B, 10% C), 51 min (95% A, 5% B, 0% C), 56 min (95% A, 5% B, 0%C), and 61 min (0% A, 5% B, 95% C), at a flow rate of 0.8 mL/min. Metabolite 3-PBA was detected at 230 nm. Under these chromatographic conditions, 3-PBA eluted at 27.1 min.

The metabolite of lambda–cyhalothrin, 3-PBA was identified by spiking the authentic compounds in the metabolic reaction sample, and by comparing with the control without substrate and the control without enzyme. Data collection and analysis were conducted using ChemStation software (Agilent Technologies, Inc., Wilmington, DE).

The quantification of 3-PBA as metabolite of lambda–cyhalothrin was conducted by using the standard curve methods. The linearity between the peak area (Y) and the concentration of 3-PBA (c, μmol/L) were investigated by using a series concentrations of 3-PBA. For 3-PBA, the linear regression equation used was Y = 24.38c+1.55, with R^2^ = 0.998.

### Data analyses and statistics

LC_50_ values were calculated by probit analysis using SPSS software. For analysis of the enzymatic activity and metabolism experiment, the data were presented as means (±*S*.*E*.) of three replicates. Difference analysis was performed by using student *t* tests with SPSS software. A value of *p*< 0.05 was considered significant.

## Results

### Lambda–cyhalothrin toxicity to *H*. *armigera* larvae and synergism assessment

The effects of quercetin intake on the sensitivity of *H*. *armigera* larvae to lambda–cyhalothrin were listed in Table 1. Lambda–cyhalothrin showed lower toxicity to the treatment group of *H*. *armigera* larvae, which had fed on the artificial diet incorporated with 0.1% (g/g artificial diet) quercetin, compared with the control group. The LC_50_ value of lambda–cyhalothrin to the treatment group was 190.83 mg/L while this value was 79.10 mg/L for the control. Despite that DEF treatment exerted low synergism to lambda*–*cyhalothrin in the control group, such a treatment effectively synergized lambda–cyhalothrin efficacy in quercetin-fed *H*. *armigera* larvae with a synergism ratio of 1.76 (Table 1).

**Table 1:** The influences of quercetin intake and synergism effect of DEF on the lambda–cyhalothrin toxicity to *H*. *armigera* larvae.

Group	N^a^	Slope±*SE*	r	LC_50_ (mg/L)	95% CL^b^	*df*	*χ*^2^	SR^c^
0.1% quercetin	175	7.21±1.20	0.98	190.83	176.51–203.56	3	0.66	-
0.1% quercetin+DEF	178	6.06±1.19	0.99	108.68	100.41–117.72	3	0.21	1.76
control	177	3.01±0.67	0.96	79.10	60.61–92.27	3	0.76	-
control +DEF	178	5.05±0.97	0.99	60.12	54.80–66.39	3	0.14	1.32

^a^N, total number of *H*. *armigera* larvae

^b^The 95% lower and upper confidence limits of LC_50_.

^c^SR, synergism ratio.

### The influence of quercetin on carboxylesterase activity in the *H*. *armigera* larvae

**Fig 2:**
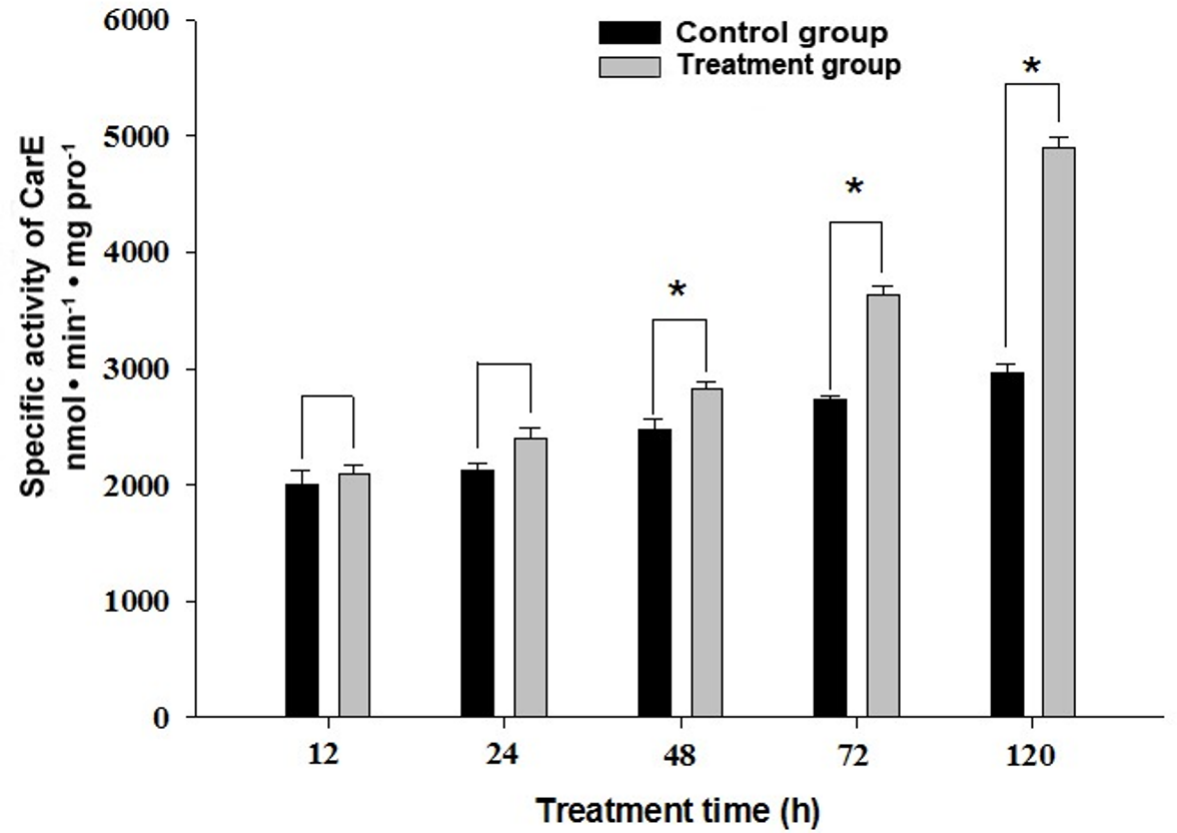
The effect of quercetin intake on carboxylesterases activity at different treatment time. Data in the figure are the mean ± *SE*. Asterisks (*) indicate significant differences within same treatment time at the 0.05 level.

The treatment group exhibited significant higher carboxylesterase activity (*p*<0.05) than the control group at 48, 72 and 120 h, respectively (Fig 2 and S1 Table). The highest CarE activity in the treatment group was observed at 120 h, which reached 1.65-fold higher than that of the control.

### In-vitro metabolism of lambda–cyhalothrin

**Fig 3:**
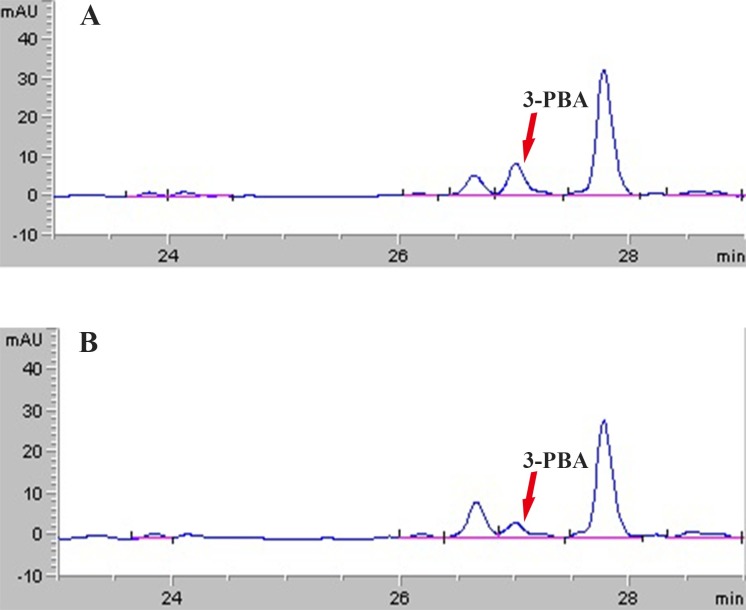
HPLC chromatograms of in-vitro hydrolytic metabolism of lambda–cyhalothrin by the crude homogenates of *H*. *armigera* larvae midguts. Metabolite 3-PBA of lambda–cyhalothrin is pointed out with arrow. (A) Indicates metabolism of lambda–cyhalothrin catalyzed by midguts homogenates from the treatment group of *H*. *armigera* larvae with 0.1% quercetin for 72 h, (B) indicates metabolism of lambda–cyhalothrin catalyzed by midguts homogenates from the control group of *H*. *armigera* larvae.

**Fig 4:**
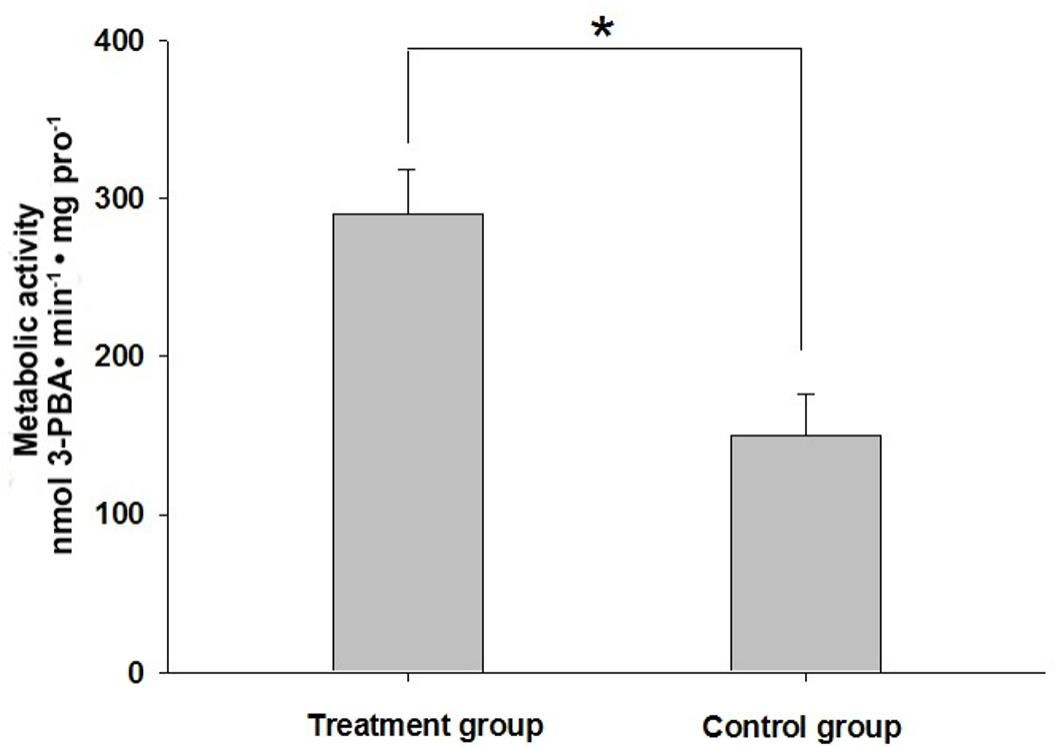
In-vitro hydrolytic metabolism of lambda–cyhalothrin by crude homogenates of *H*. *armigera* larvae midguts. Asterisks (*) indicate significantly different between treatment and the control (untreated) group at the 0.05 level.

The metabolite 3-PBA of lambda–cyhalothrin was markedly detected with the retention time at 27.1 min (Fig 3). Moreover, the hydrolytic metabolism activity was significantly induced after *H*. *armigera* larvae fed on quercetin for 48 h (Fig 4). The hydrolytic metabolic activity to lambda–cyhalothrin was 289.82±28.59 nmol 3-PBA/mg protein/min for the treatment group while this value was 149.60±26.90 nmol 3-PBA/mg protein/min for the control.

## Discussion

Our data demonstrated that the *H*. *armigera* larvae which fed on artificial diets incorporated with 0.1% quercetin exhibited higher tolerance to lambda–cyhalothrin than the larvae that feed on the diet without quercetin. This finding enriched the growing body of literatures showing that plant secondary metabolites in host plant could affect insecticide sensitivity of herbivorous insects. Such a hypothesis may apply to the insects from various feeding guilds. For example, after being exposed to xanthotoxin, *Helicoverpa zea* caterpillars displayed enhanced tolerance to *α*-cypermethrin (16% mortality) in comparison with the control caterpillars (40% mortality) [32]. Long-term induction of host plants for B-biotype *Bemisia tabaci* also influenced their susceptibilities to several insecticides [33]. Quercetin showed antagonistic effect to Cry1Ac toxicity in *H*. *armigera* [34]. In addition, incorporation of quercetin into diet significantly enhanced the tolerance of bees *Apis mellifera* to tau-fluvalinate [35].

One of the mechanisms underlying the impacts of secondary metabolites on insect susceptibility to insecticide is the role of detoxification system in the insect. CarE is one of the major classes of detoxification enzymes involved in detoxification of xenobiotics including plant secondary metabolite and insecticides. In this study, we found that the tolerance to lambda–cyhalothrin in quercetin-fed *H*. *armigera* larvae was associated with the quercetin induced elevation of CarE enzyme activity. The CarE activity in *H*. *armigera* larvae midgut showed a significant increase after they fed on 0.1% quercetin for 48 h (Fig 2), in addition to the increased tolerance to lambda–cyhalothrin of quercetin-fed *H*. *armigera* larvae. Moreover, the synergist DEF showed significant synergism effect on the toxicity of lambda-cyhalothrin to the quercetin-fed *H*. *armigera* (Table 1). Several previous studies also showed that CarE activity in insects could be affected after plant secondary metabolites exposure. For example, the CarE activity in *H*. *armigera* was significant induced after they were fed with rutin, quercetin or 2- tridecanone for several generations [36]. Specific CarE activity was also significantly induced in *H*. *armigera* treated with methyl jasmonate [37].

Despite the potential role of CarE enzyme activity in *H*. *armigera* physiology, the contribution of the increased detoxification enzyme activity to the detoxification metabolic capacity of insecticide is still unknown. Therefore, we further explored the influences of quercetin intake on the in-vitro lambda-cyhalothrin hydrolytic metabolism by *H*. *armigera* larvae midgut homogenates. As expected, we found quercetin intake by *H*. *armigera* larvae induced more production of nontoxic metabolite 3-PBA from hydrolysis of lambda–cyhalothrin, and this indicated that quercetin-fed *H*. *armigera* larvae midgut exhibited more hydrolytic metabolic capacity to lambda–cyhalothrin, compared to the control. It is well-known that CarE mediated hydrolytic metabolism contributed to the cleavage of ester linkage of pyrethroids to give acid moiety and alcohol moiety 3-phenoxy-benzylalcohol (3-PBAlc). 3-PBAlc was further oxidized into 3-phenoxybenzylaldehyde (3-PBAld), 3-PBA, and 4′-OH-3-PBA [38–41]. Therefore, the elevated CarE activity in quercetin-fed *H*. *armigera* larvae could hydrolyze more lambda-cyhalothrin and generate more 3-PBA in the treatment group finally.

Though clear evidences were obtained on the elevated hydrolytic metabolic capacity mediated by CarE resulted in enhanced tolerance to lambda-cyhalothrin in quercetin-fed *H*. *armigera*. The detoxification metabolism of lambda-cyhalothrin through P450s and GSTs may also contribute to the lambda-cyhalothrin tolerance in quercetin-fed *H*. *armigera*, because the metabolic pathways of pyrethroids in insects also include the P450s-mediated oxidation and the conjugation of pyrethroids metabolites to GSH catalyzed by GSTs [42–46]. Therefore, the contribution of P450s and GSTs to lambda-cyhalothrin tolerance after quercetin intake needs to be investigated in further works, with the aim to fully understand the roles of detoxification enzyme system of insects played in adaption to host plant and insecticides tolerance.

Based on the results in this study, exposure of *H*. *armigera* larvae to quercetin could deteriorate pyrethroid insecticides toxicity. Thus, special caution needs to be taken when spraying lambda-cyhalothrin in the quercetin-rich crops, since the management of *H*. *armigera* may fail. Be unaware of this reason, the farmers may readily carry out repeated applications of pyrethroid insecticides which are not only costly and labor-intensive, but also pose significant risks to non-target beneficial arthropods in the field. More broadly, if the adjacent non-crop plants are rich in quercetin or other secondary metabolites which could help *H*. *armigera* to “resist” the insecticide toxicity, then the IPM programs should consider removing those non-crop plants surrounding crop fields. At the same time, this phenomenon could also provide valuable information on transgenic plant. Through interfering key detoxification enzyme gene which responds to both plant secondary metabolites and insecticides by expressing double-stranded RNA, it is possible to reduce the adverse effects of plant secondary metabolites on insecticidal effects.

In summary, the enhanced hydrolytic metabolism capacity of lambda–cyhalothrin after quercetin intake resulted in efficiently detoxification metabolism of lambda–cyhalothrin, thus enhanced the tolerance to lambda–cyhalothrin of *H*. *armigera* larvae. As a result, the elevated carboxylesterase activity contributes to lambda-cyhalothrin insensitivity in quercetin fed *H*. *armigera*larvae.

## Supporting information

S1 Table The effects of quercetin intake on carboxylesterases activity at different treatment time. Asterisks (*) indicate significant differences within same treatment time.
(PDF)
