# Supplementary material for: Elevated carboxylesterase activity contributes to the lambda-cyhalothrin insensitivity in quercetin fed Helicoverpa armigera (Hübner)
Source: PLoS One. 2017 Aug 17;12(8):e0183111. doi: 10.1371/journal.pone.0183111 (PMC5560706; doi:10.1371/journal.pone.0183111)
Supplement: S1 Table — Asterisks (*) indicate significant differences within same treatment time. (PDF) [file pone.0183111.s001.pdf]

Supporting information:

Table S1 The effects of quercetin intake on carboxylesterases activity at different treatment time

Treatment time (h)	specific activity of CarE (nmol ·min^{-1} ·mg pro^{-1})		*P* vaule
	Treatment group (mean ± *SE*)	Control group (mean ± *SE*)	
12	2088.10 ±85.75	2001.57 ±122.82	0.59
24	2391.42 ±100.77	2130.08 ±52.61	0.08
48	2818.14 ±61.04	2476.35 ±83.77	0.03*
72	3637.06 ±70.97	2731.57 ±30.40	0.00*
120	4896.04 ±95.53	2962.57 ±71.24	0.00*

Asterisks (*) indicate significant differences within same treatment time.
